# *N*-Palmitoylethanolamine Prevents the Run-down of Amplitudes in Cortical Spreading Depression Possibly Implicating Proinflammatory Cytokine Release

**DOI:** 10.1038/srep23481

**Published:** 2016-03-23

**Authors:** Frank Richter, Peter Koulen, Simon Kaja

**Affiliations:** 1 Institute of Physiology I/Neurophysiology, Jena University Hospital-Friedrich Schiller University Jena, Jena, Germany; 2 Vision Research Center, Department of Ophthalmology, University of Missouri – Kansas City, School of Medicine, 2411 Holmes St., Kansas City, MO 64108, USA; 3 Department of Basic Medical Science, University of Missouri – Kansas City, School of Medicine, 2411 Holmes St., Kansas City, MO 64108, USA; 4 Departments of Ophthalmology and Molecular Pharmacology and Therapeutics, Loyola University Chicago, Stritch School of Medicine, 2160 South First Ave., Maywood, IL 60153, USA; 5 Edward Hines Jr. VA Hospital, Research Service, 5000 S Fifth Ave., Hines, IL 60141, USA

## Abstract

Cortical spreading depression (CSD), a wave of neuronal depolarization in the cerebral cortex following traumatic brain injury or cerebral ischemia, significantly aggravates brain damage. Here, we tested whether *N*-palmitoylethanolamine (PEA), a substance that effectively reduces lesion volumes and neurological deficits after ischemic stroke, influences CSD. CSD was elicited chemically in adult rats and occurrence, amplitude, duration and propagation velocity of CSD was determined prior to and for 6 hours after intraperitoneal injection of PEA. The chosen systemic administration of PEA stabilized the amplitude of CSD for at least four hours and prevented the run-down of amplitudes that is typically observed and was also seen in untreated controls. The propagation velocity of the CSD waves was unaltered indicating stable neuronal excitability. The stabilization of CSD amplitudes by PEA indicates that inhibition or prevention of CSD does not underlie PEA’s profound neuroprotective effect. Rather, PEA likely inhibits proinflammatory cytokine release thereby preventing the run-down of CSD amplitudes. This contribution of PEA to the maintenance of neuronal excitability in healthy tissue during CSD potentially adds to neuroprotection outside a damaged area, while other mechanisms control PEA-mediated neuroprotection in damaged tissue resulting from traumatic brain injury or cerebral ischemia.

Stroke is the fourth leading cause of death in the United States, accounting for 1 in 20 deaths^1^. Approximately 795,000 stroke cases are reported every year, making it a significant health problem^1^.

In malignant hemispheric stroke, two areas of injury within the ischemic cerebrovascular bed can be distinguished: the ischemic core and the penumbra. The penumbra is characterized by hypoperfusion and electrical function insufficient to sustain neuronal function, but adequate to maintain cellular survival^2^. As such, the penumbra represents the primary target in efforts to develop novel pharmaceutical strategies against stroke and for the prevention of secondary damage after stroke^3^. One such sequelae of stroke is cortical spreading depression (CSD), a depolarization wave in the cerebral gray matter, originally described by Leao^4^. In addition, the higher prevalence of stroke in patients suffering with migraine^5^,^6^, where CSD has been identified as a characteristic hallmark^7^ , broadens the clinical rationale to study the role of CSD in stroke. Depolarizations resembling CSD were identified in the penumbra region of experimentally induced lesions and termed peri-infact depolarization (PID)^8^,^9^. These spontaneous depolarizations share the characteristic features of experimentally-induced CSD, including a shift in plasma membrane polarization, disturbed ion homeostasis, and altered propagation velocities^7^,^8^. Despite this preclinical evidence the pathologic role and mechanistic involvement of CSD in neurological disorders and traumatic brain injury has been ignored in clinical practice until recently, when events resembling both PID and CSD were identified in stroke patients by the Co-Operative Studies on Brain Injury Depolarizations consortium^10^.

We have previously described the neuroprotective effects of *N*-acylethanolamines in a rat model of ischemic stroke using temporary middle cerebral artery occlusion (MCAO) to generate ischemia/reperfusion injury^11^,^12^. *N*-Palmitoylethanolamine (PEA) belongs to the group of *N*-acylethanolamines (NAEs), and is an endogenous signaling lipid involved in cellular signaling and neuroprotection^13^,^14^,^15^,^16^. While several NAEs, such as anandamide, are ligands for cannabinoid receptors, we and others have shown that the neuroprotective effects of PEA are mediated by an intracellular mechanism independent of cannabinoid and vanilloid receptor activation^11^,^12^,^14^,^16^,^17^,^18^.

Here, we investigated whether the neuroprotective properties of PEA mediated by intracellular signaling pathways and resulting in reduced lesion size and improved neurological deficit score after experimental stroke, also contribute mechanistically to CSD/PID events and their deleterious impact on stroke.

## Methods

### Animals

The *in vivo* experiments in the present study were approved by the Thuringian Government (Registration Numbers 02-040/06 and 02-005/12) and performed according to the Protection of Animals Act of the Federal Republic of Germany. The animals were treated in strict adherence to the American Physiological Society’s Guiding Principles in the Care and Use of Vertebrate Animals in Research and Training.

### Surgical preparation of the rats

Adult male Wistar rats (n = 9, 350–450 grams) were deeply anesthetized with sodium thiopental (Trapanal^®^, Inresa GmbH, Freiburg, Germany; initially 100 mg/kg i.p.). During dissection depth of anesthesia was checked by testing the corneal blink reflex and reflexes to noxious squeezing the tail tip. During the experiments, absence of the corneal blink reflex was maintained by supplemental doses of 20 mg/kg i.p. The trachea, the right femoral vein and artery were cannulated. The mean arterial blood pressure and the electrocardiogram were continuously monitored. Body temperature was kept at 37 °C by a feedback controlled heating system.

After stereotactic fixation of the head a trephination was made over the left hemisphere (spanning from the coronal suture over a length of 5–8 mm, 3–4 mm wide) using a mini-drill under cooling with artificial cerebrospinal fluid (ACSF, in mmol/L: NaCl 138.4, KCl 3.0, CaCl_2_ 1.3, MgCl_2_ 0.5, NaH_2_PO_4_ 0.5, urea 2.2, glucose 3.4, warmed to 37 °C and equilibrated with 5% CO_2_ in O_2_). The underlying dura and arachnoidea were removed; the exposed cortex was kept moist with ACSF. A wall was built with dental acrylic on the skull around the trephination thereby forming a trough in which the exposed cortex was superfused with ACSF.

### Recording of intracortical DC potentials

**Figure 1:**
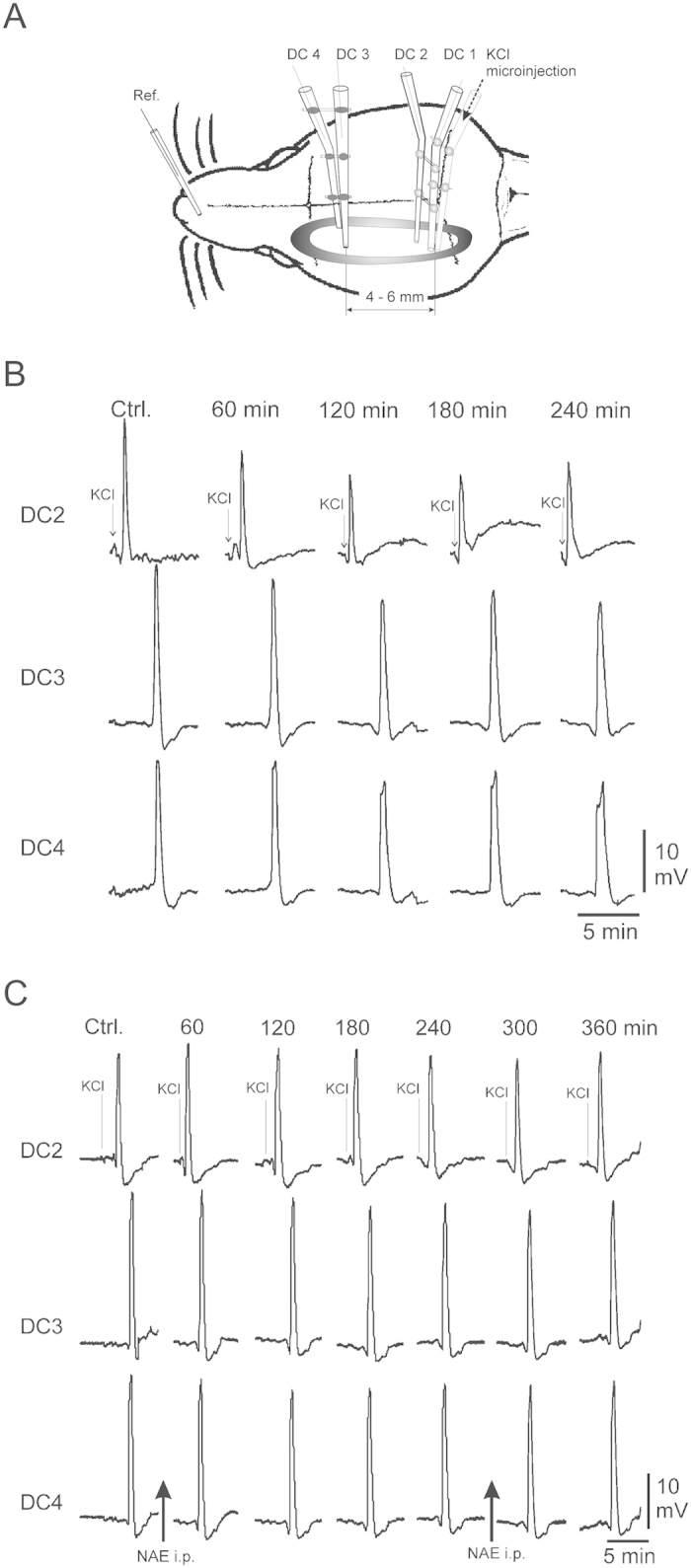
Display of experimental approach and effect of PEA on CSD. (**A**) Schematic view of the adult rat skull (not to scale) with the trephination that is surrounded by a wall made from dental acrylic (grey oval). Electrode arrays were lowered to a depth of 1,200–1,400 μm, approximately cortical layer V for the deepest electrode tips. Recording electrodes in an array had vertical and horizontal tip separations of 800 μm. (**B**) Representative samples of CSD from electrode DC2, DC3, and frontal electrode DC 4 in adult rat cortex when only ACSF was applied to the cortical surface and no PEA was given. Arrows mark the microinjection of KCl. (**C**) CSDs in cerebral cortex before and after intraperitoneal application of PEA. Arrows mark the microinjection of KCl.

We used an Ag/AgCl reference electrode (containing 2 M KCl) on the nasal bone. The electrode arrays for elicitation of CSD and recordings of intracortical DC potentials at sites 1–4 are displayed in Fig. 1A. Electrodes for DC recordings had tip diameters of about 5 μm, resistance <10 MΩ, and were filled with 150 mmol/L NaCl. The distance between the electrode tips was 400–500 μm. The electrode for CSD elicitation contained 1 mol/L KCl; SDs were elicited by injection of 0.5 μL KCl with a pressure of 100 kPa for 1 second using a microinjector (picoinjector PLI-100; Harvard Apparatus, Holliston, MA, USA). The signals were recorded using a four-channel high-impedance amplifier (Meyer, Munich, Germany) and stored on PC. CSDs were accepted if they had a steep onset, exceeded amplitudes ≥5 mV, and migrated over the whole recording area. At the end of an experiment the animals were euthanized by an overdose of the anesthetic (100 mg per rat, intravenously).

PEA was dissolved in ethyl alcohol and administered intraperitoneally at a dose of 20 mg/kg body weight. The recording protocol consisted of at least two CSD elicitations at intervals of 30 min prior to the injection of PEA forming a baseline. After injection of PEA, CSD were elicited at intervals of 30 min for two hours and then every hour. Four hours after the injection the rats received a second dose of PEA (20 mg/kg body weight), and CSD elicitations were continued 30 min, one hour and two hours after the second injection.

In a subset of three adult rats, two instead of one trephinations were made over the left hemisphere (1 frontal, spanning from 2 mm in front of bregma over a length of 5–6 mm, 3–4 mm wide, and 1 caudal, with a diameter of 3–4 mm in front of lambda), and a wall was built with dental acrylic on the skull around the frontal trephination thereby forming a trough with a capacity of 150 to 200 μL. The same sets of recording electrodes (DC 1 and DC 2 in the untreated area; DC 3 and DC 4 in the treated area) and the same stimulation device as in the experiments with PEA were used.

After testing for CSD under control conditions (both threphinations were superfused with warmed equilibrated ACSF), in the trough around the frontal trephination 1 μg of the IL-1ß receptor antagonist Anakinra (recombinant N2-L-methionyl-interleukin 1 receptor antagonist; Sobi, Stockholm, Sweden) was applied until the end of the experiment. CSD were elicited following the same schedule as in the experiments with PEA. CSD amplitudes in untreated and treated areas were evaluated and CSD propagation velocity was calculated from the time interval between microinjection of KCl and the peak of the CSD in the treated area.

Data are reported as means ± S.E.M. Statistical analysis was performed using the Wilcoxon matched pairs signed rank test. Bonferroni adjustment was performed as necessary. Significance was accepted at P < 0.05.

## Results

### *In vivo* CSD recordings

Microinjection of KCl elicited single propagating CSD with typical parameters (cf. Somjen)^19^ in all rats at all recording electrodes. Typically observed delays between the DC potential peaks (as shown in Fig. 1B,C, traces DC2 and DC3/DC4), are due to the distance between the recording electrode arrays.

**Figure 2:**
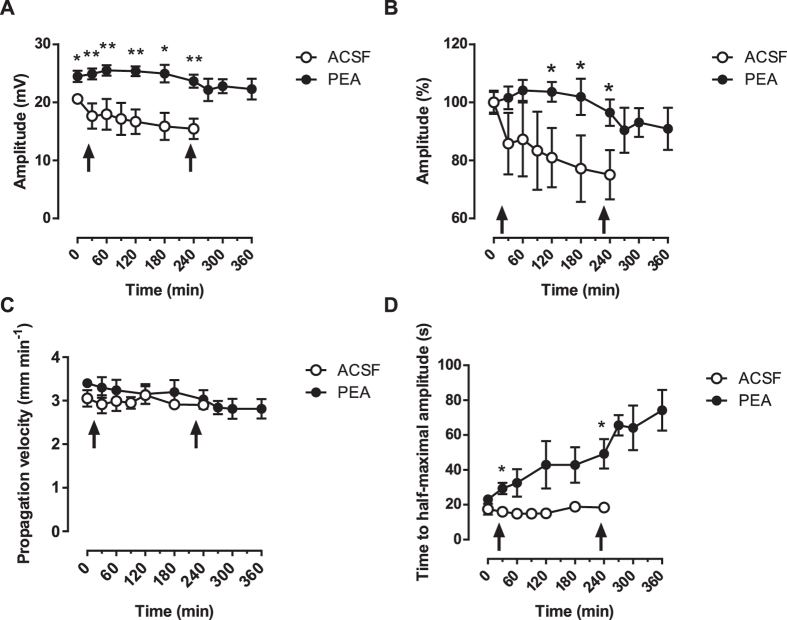
Comparison of the effect of PEA on different parameters of CSD. Empty circles represent vehicle-treated animals, filled circles animals treated with PEA. The arrows on the abscissae indicate i.p. injections of either vehicle or PEA. Data are presented as mean values ± S.E.M. (**A**) Comparison of amplitudes of CSD in untreated animals vs. treated animals (*p < 0.05; **p < 0.01); (**B**) Normalized change in CSD amplitudes in untreated animals vs. treated animals (*p < 0.05); (**C**) Comparison of propagation velocity of CSD in untreated animals vs. treated animals; (**D**) Comparison of CSD duration at half maximal amplitude in untreated animals vs. treated animals (*p < 0.05).

In rats that were not dosed with PEA (vehicle control animals), we observed a continuous decline of CSD amplitudes at all three recording sites (Fig. 1B). At the end of the experiments after four hours, we were still able to elicit CSD with similar slopes, but CSD amplitudes were reduced significantly to 79.5 ± 2.7% of amplitudes at the beginning of the experiments (Fig. 2A,B; n = 3; P < 0.05). In parallel, the propagation velocity of the experimentally induced CSD remained unaltered (2.3 ± 0.1 mm/min at the beginning versus 2.4 ± 0.2 mm/min at the end of experiments (Fig. 2C; n = 3; P = 0.664), i.e. a decrease of 4.3 ± 8.1%), while CSD duration at half maximal amplitude increased by 27.7 ± 35.6%, from 19.5 ± 3.7 s to 24.9 ± 8.6 s (Fig. 2D; n = 3, P = 0.240).

In contrast in PEA-treated rats, amplitudes of the experimentally induced CSD remained constant at all recording sites over the entire experimental observation period (Fig. 1C) with a statistically not significant decline to 92.1 ± 2.0% of baseline after four hours (Fig. 2A,B; n = 5; P = 0.262). After a second systemic dosing with PEA this statistically not significant decrease continued, but reached only 83.6 ± 6.4% of the amplitudes at the beginning of the experiment two hours after that (Fig. 2A,B; n = 5; P = 0.283). In the same time, CSD propagation velocity was unchanged statistically with a decline from 3.4 ± 0.3 mm/min to 3.0 ± 0.5 mm/min in the first four hours after the first application of PEA (Fig. 2C; n = 5; P = 0.321) and to 2.8 ± 0.5 mm/min in the two hours after the second injection with PEA (Fig. 2C; n = 5; P = 0.167). CSD duration at half maximal amplitude increased significantly from 23.12 ± 1.2 s to 49.2 ± 8.5 s (Fig. 2D; n = 5; P < 0.05) in the first four hours. In the two hours after the second injection with PEA, CSD duration at half maximal amplitude further increased significantly to 74.3 ± 11.8 s (Fig. 2D).

**Figure 3:**
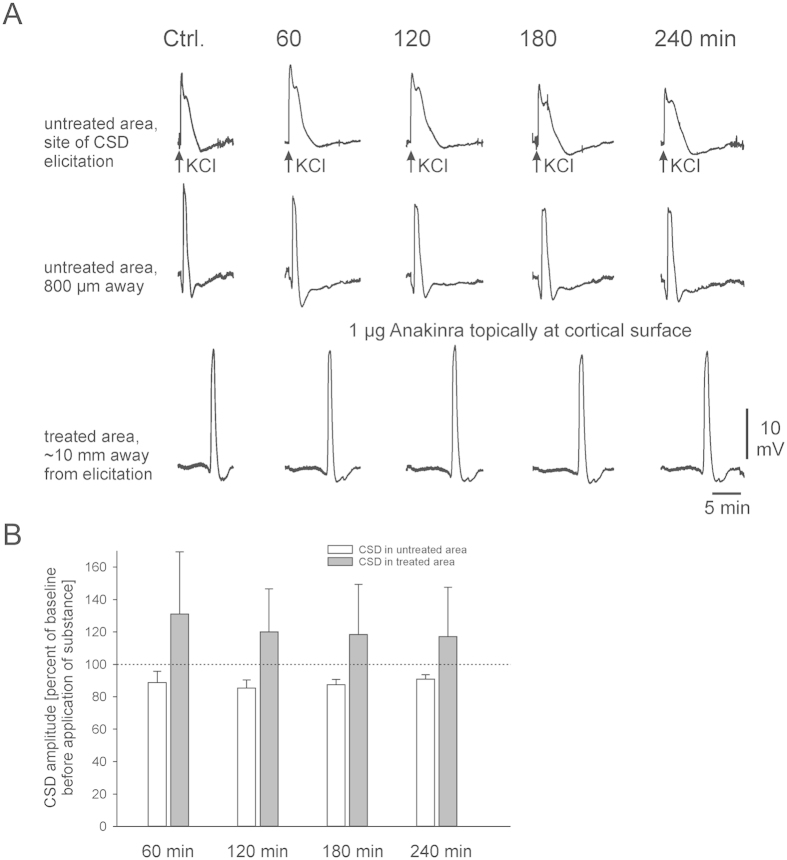
Blockade of IL-1β receptors prevents CSD amplitude rundown. (**A**) Representative samples of CSD in adult rat cortex in the untreated area from electrode DC 1 at the site of KCl microinjection, and electrode DC 2 (about 800 μm away) and from electrode DC 3 in the treated area (about 10 mm away from CSD elicitation) before and after application of 1 μg Anakinra. (**B**) Superfusion of a brain area with Anakinra prevents the effect of IL-1ß on CSD, stabilizing CSD amplitudes. Bars represent mean CSD amplitudes ± S.E.M. at different time points with topical application of Anakinra (n = 3).

In a subset of experiments, we tested the effects of the IL-1β blocker, Anakinra. As evident from the sample traces (Fig. 3A), CSD amplitudes slowly decreased to 90.1 ± 2.8% of control in the untreated cortical area. In the treated area, however, such decrease did not occur and CSD amplitudes were stable. Summary data (Fig. 3B) indicate that blockade of IL-1ß receptors stabilizes CSD amplitudes, confirming that IL-1ß contributes to the run-down of CSD amplitudes in the cortical area superfused with ACSF. The slight increase in CSD amplitudes in the untreated area 240 min after beginning of the treatment might be due to diffusion of the drug below the skull and a drug effect in remote brain areas as well.

## Discussion

The *in vivo* study showed that systemically applied PEA is able to keep the amplitude of single experimentally induced CSD events at a stable level for at least four hours, whereas in untreated controls, we measured a significant run-down of CSD amplitudes in the same time interval. In parallel, CSD propagation velocity was unaltered and the lengthening of CSD duration at half maximal amplitude was the same in controls and PEA-treated animals.

This novel finding that dosing with PEA prevents the run-down of CSD amplitudes differs from all observations on CSD in untreated control animals or untreated cortical areas that were used for example to determine the effects of topically applied cytokines^20^.

An inflammatory process in the exposed cortex of the healthy rat could be a reason for the rundown of the CSD amplitudes in the native control animals. Though the cortex surface in our experiments was superfused with warmed, equilibrated ACSF, this cannot prevent an activation of the microglia as a source of cytokines. In previous work we have shown, that the proinflammatory cytokine TNFα dose-dependently diminishes amplitudes of CSD^20^. TNFα is released after brain trauma (TBI) or after stroke^21^,^22^,^23^,^24^,^25^. Similarly, the proinflammatory cytokine IL-1ß is able to diminish CSD amplitudes^26^ and also released after TBI and/or stroke^22^,^27^.

It has been shown previously that induction of repetitive CSD by KCl results in a 24-fold increase in IL-1β mRNA ipsilaterally within a 4 hr time window^28^. Importantly, such increase could not be induced by other stimuli, and was predominantly localized to ramified microglia in the cortex^28^. Our results provide evidence that blockade of the IL-1β receptor using Anakinra prevents CSD amplitude rundown in our experimental paradigm (Fig. 3), directly implicating cytokine signaling as the underlying physiological mechanism. There is evidence from the literature, that activation and secretion of IL-1ß is mediated via the inflammasome^29^,^30^ which is formed from intracellular protein complexes that activate caspases needed to convert pro IL-1ß into bioactive IL-1ß^31^,^32^. The activators of the inflammasome are potassium efflux^33^ and/or decrease of the intracellular potassium concentration either caused by the potassium efflux itself or by the influx of water from the extracellular space together with an influx of sodium ions^34^. All three elements needed for activation of the inflammasome occur during CSD: potassium ions transiently leave the neurons and glial cells, and in the same time sodium ions and water enter the cells^35^, leading to shrinkage of the extracellular space down to 35% of the normal values^36^. It is likely therefore, that activation of the inflammasome by CSD is the reason for a release of IL-1ß causing the decline in CSD-amplitudes. Indeed, this decrease was prevented, when the IL-1ß pathway was blocked by Anakinra.

Several previous reports have shown reduced levels of IL-1β following PEA administration. For example, Di Paola and colleagues measured significantly reduced IL-1β levels after PEA in their splanchnic artery occlusion (SAO) shock model^37^. Similarly, intrathecal administration of PEA attenuated the increase of IL-1β levels in the rat spinal cord^38^, and analogously, the fatty acid amide hydrolase (FAAH) inhibitor URB597 resulted in an up-regulation of endogenous PEA and an attenuation of induction of IL-1β in a model for Toll-like receptor 3-mediated neuroinflammation^39^. Altogether, it appears logical to speculate that PEA treatment counteracts the upregulation of IL-1β during CSD, thereby stabilizing CSD amplitudes.

Our previous studies have excluded cannabinoid receptors as mediators of PEA’s neuroprotective properties in a preclinical model for stroke^11^,^12^,^14^,^16^. It has been established that activation of CB1 receptors by WIN 55212-2 dose-dependently suppressed CSD amplitude, duration and propagation velocity^40^. The same compound also decreased the spatial spread of neocortical excitation by interfering with glutamatergic neurotransmission in an *in vitro* assay^41^. By contrast, local application of WIN 55212-2 together with the AMPA receptor blocker CNQX facilitated the propagation of CSD in rat brain slices from neocortex into the hippocampus^42^. Therefore, a CB1 receptor-mediated mechanism underlying the prevention of a run-down of CSD amplitudes by PEA is unlikely.

Another potential mechanism for the action of PEA is a reduction of CSD-induced astrogliosis and reduced astrocyte-mediated cytokine release. It has previously been shown that potassium concentrations, similar to those occurring during CSD, result in reactive astrogliosis in the rat cortex^43^, hippocampus^44^, and spinal cord^45^. This research served as the basis for our own previous work, in which we concluded that changes in the extracellular space volume and geometry after CSD are the result of astrogliosis^36^. Similarly, astrogliosis has more recently been described after status epilepticus^46^, which shares many aspects of hyperexcitability with acute CSD. Ahmad and colleagues^47^ have reported that PEA blocked the infiltration of astrocytes after ischemic brain injury in mice and inhibited the expression of pJNK, NF-κB and degradation of IκB-α^47^. Signaling pathways controlled by these three mediators are linked to the release of proinflammatory cytokines and/or to the control of their actions after receptor binding.

In summary, our data support the notion that systemic application of PEA in our experimental paradigm prevented or reduced the release of proinflammatory cytokines. Such cytokines are released in our experimental model as a consequence of trephination of the skull and exposure of the brain surface, leading to a slow rundown of the CSD amplitude.

## Additional Information

**How to cite this article**: Richter, F. *et al.* *N*-Palmitoylethanolamine Prevents the Run-down of Amplitudes in Cortical Spreading Depression Possibly Implicating Proinflammatory Cytokine Release. *Sci. Rep.* **6**, 23481; doi: 10.1038/srep23481 (2016).
